# Responses in Growth and Anatomical Traits of Two Subtropical Tree Species to Nitrogen Addition, Drought, and Their Interactions

**DOI:** 10.3389/fpls.2021.709510

**Published:** 2021-08-02

**Authors:** Yiyong Li, Zhaocheng Wang, Huihui Liu, Cheng Zhang, Songling Fu, Xiong Fang

**Affiliations:** ^1^School of Forestry and Landscape Architecture, Anhui Agricultural University, Hefei, China; ^2^Hefei Urban Ecosystem Research Station, National Forestry and Grassland Administration, Hefei, China; ^3^College of Land Resources and Environment, Jiangxi Agricultural University, Nanchang, China; ^4^College of Resources and Environment, Fujian Agricultural and Forestry University, Fuzhou, China

**Keywords:** water deficit, leguminous, wood anatomy, leaf anatomy, non-structural carbohydrate

## Abstract

Nitrogen (N) deposition and drought are two major stressors that influence tree growth and propagation. However, few studies have investigated their interactions. In this study, saplings of the two co-occurring species *Ormosia pinnata* (leguminous) and *Schima superba* (non-leguminous) were cultivated under two N addition rates (0 and 80 kg N ha^–1^ year^–1^) with well-watered (WW, 80% of field capacity), moderate drought (MD, 60% of field capacity), and severe drought conditions (SD, 40% of field capacity). We examined their growth, as well as multiple anatomical and non-structural carbohydrate (NSC) responses, after 2 years. Results revealed that N addition significantly promoted the growth of MD-stressed *S. superba*, whereas no significant effect was detected in *O. pinnata*. Decreased leaf water potential (both Ψ_md_ and Ψ_pd_) was also observed with N addition for both species under MD, but not under SD. Furthermore, the application of N positively impacted drought adaptive responses in the stem xylem of *S. superba*, showing decreased stem xylem vessel diameter (*D*_H_), theoretical hydraulic conductivity (*K*_th_), and increased vessel frequency (*VF*) upon drought under N addition; such impacts were not observed in *O. pinnata*. Regarding leaf anatomy, N addition also caused drought-stressed *S. superba* to generate leaves with a lower density of veins (*VD*) and stomata (*SD*), which potentially contributed to an enhanced acclimation to drought. However, the same factors led to a decrease in the palisade mesophyll thickness (*PMT*) of SD*-*stressed *O. pinnata*. Moreover, N addition increased the xylem soluble sugar and starch of MD-stressed *O. pinnata*, and decreased the xylem soluble sugar under SD for both species. The results suggest that N addition does not consistently modify tree growth and anatomical traits under variable water availability. *S. superba* appeared to have a greater capacity to be more adaptable under the future interactive effects of N addition and drought due to major modifications in its anatomical traits.

## Introduction

Atmospheric nitrogen (N) deposition and drought are two major stressors that influence the structures, functionalities, and services of forest ecosystems (Wamelink et al., 2009; Ma et al., 2012; Garcia-Valdes et al., 2021). The frequency and intensity of seasonal and periodic droughts are escalating globally, causing an increased decline of forests in many regions (Allen et al., 2010; Senf et al., 2020). Increased N deposition often stimulates tree growth (Xia and Wan, 2008), which can, in turn, alter the drought adaptation properties of plants (Pivovaroff et al., 2016; Shi et al., 2020) and affect drought-induced mortality. However, there is no clear consensus on the magnitude and trajectory of this effect and how it interacts with the impacts of drought on tree species. Given the assumption that a drier and N-enriched environment is likely to occur in the future, an improved elucidation of how tree species cope with combined effects of N deposition and drought will be essential for improving predictions regarding the functioning of forest ecosystems.

Nitrogen and water are essential for plant growth. N addition can stimulate plant photosynthesis and growth when other resources are not limited (Zhao and Zeng, 2019; Liang et al., 2020), but excessive N inputs may also lead to a reduction in growth (Mo et al., 2008). In contrast, drought stress typically limits plant photosynthesis, resulting in lower production of biomass (Yan et al., 2016). Previous studies have highlighted that, in some cases, the mitigation of N addition in drought-stressed plants induced inhibitory effects by improving drought resistance (Ibanez et al., 2018; Zhang et al., 2018) and avoiding “carbon starvation” (Tarvainen and Näsholm, 2017). Conversely, other studies have suggested that N addition has a “fertilization effect” on plant growth, which increases evaporative consumption and water demands, thereby increasing drought sensitivity (Meyer-Grünefeldt et al., 2015; Dziedek et al., 2016). These conflicting results reflect the need to improve the mechanistic understandings of the authors on how N addition and drought would interactively affect the physiological performance of trees. Of the different characteristics that have been linked to tree performance under changing environmental conditions, the storage of non-structural carbohydrates (NSC) can influence plant metabolism and maintain tissue water potential as osmolytes (Sala et al., 2012), which suggests that NSC could enhance drought resistance (O’Brien et al., 2014; Zhang et al., 2021). However, little is known in regard to the combined effects of drought and N deposition on the variations in the NSC of trees.

The anatomical traits of the xylem and leaves exhibit a certain degree of plasticity in response to various environmental factors, such as temperature (Castagneri et al., 2017), nutrients (Moreira et al., 2015), and water (Tng et al., 2018). Employing a meta-analysis approach, Zhang et al. (2018) found that N deposition led to an increased xylem conduit diameter, stem-specific hydraulic conductivity, and P_50_ (water potential at 50% of conductivity lost), which increased the susceptibility of plants to drought-induced hydraulic failure. Another meta-analysis by Borghetti et al. (2017) reported that N deposition led to higher vessel density, which enhanced the capacity of the xylem to withstand the risk of embolism. Previous studies found different hydraulic responses between leaves and stems/branches to N deposition and drought (Wang et al., 2016; Jin et al., 2020). While the effects of N addition and drought on xylem traits have garnered considerable attention, few studies have set their focus on the anatomical traits of leaves. This is surprising, given the fact that leaf veins, stomatal length, and density, and other leaf anatomical characteristics are regulated by many environmental factors (such as nutrients and water), and are intimately associated with the physiological performance of leaves (Tian et al., 2016; Cruz et al., 2019).

In East Asia, due to monsoonal effects, tropical and subtropical tree species suffer from seasonal drought events, the impacts of which may be intensified as a result of global climate change (Sheffield and Wood, 2008). Moreover, due to rapid economic development, naturally N-rich tropical forests in Asia receive the highest overall levels of N deposition compared to other forest biomes (Schwede et al., 2018). However, few studies have analyzed the combined influences of N inputs and drought on tropical and subtropical tree species. Leguminous tree species are particularly abundant in tropical forests (Hedin et al., 2009), representing a unique plant functional group in forests due to their potential for symbiotic N fixation (Barea et al., 1987). Previous studies have demonstrated that leguminous trees may be more sensitive to drought than non-leguminous tree species due to their unique N acquisition strategy (Minucci et al., 2019). However, to the best of our knowledge, there are little available data on whether the combined effects of N addition and drought on physiological performance can shift between leguminous and non-leguminous tree species.

In this study, we grew leguminous *Ormosia pinnata* and non-leguminous *Schima superba* saplings in a greenhouse under three watering regimes and two N addition rates for 2 years. We investigated the physiological and anatomical traits correlated to plant growth and water relations at the end of the cultivation. Specifically, we tested the following hypotheses: (1) the leguminous species *O. pinnata* is less sensitive to N supplies but more sensitive to drought than the non-leguminous species *S. superba*, and (2) N deposition can intensify the limitations of drought stress in both species.

## Materials and Methods

### Experimental Design

This experiment was conducted at the Fujian Agricultural and Forestry University, located in Fujian Province, China (26°5′9″N, 119°14′19″’E). The area has a subtropical monsoon climate, with mean annual precipitation and temperature at 1,700 mm and 21.5°C, respectively. Two evergreen broadleaved tree species, *Ormosia pinnata* (Lour.) Merr. and *Schima superba* Gardn. et Champ., were selected for the present study. *O. pinnata* is a leguminous species, whereas *S. superba* is a non-leguminous tree species. Both are dominant species that are widely distributed across subtropical evergreen broadleaved forests in China.

The experiment consisted of 18 square plots (1 × 1 × 0.6 m; length × width × depth) in a greenhouse. At the bottom of each plot, there was a drainage hole (2 cm in diameter) connected to a polyvinyl chloride tube. In November 2017, two different soil layers (0–25 and 25–50 cm) were collected from a nearby evergreen broadleaved forest and mixed separately; then placed into each plot correspondingly. The soil pH, NH_4_^+^-N, and NO_3_^–^-N were 6.09 ± 0.08, 5.62 ± 0.77, and 2.20 ± 0.51 mg kg^–1^, respectively. In December 2017, the local ecotypes of all the native saplings, which were in their second year of growth, were transplanted into the plots (one individual per species per plot).

**FIGURE 1:**
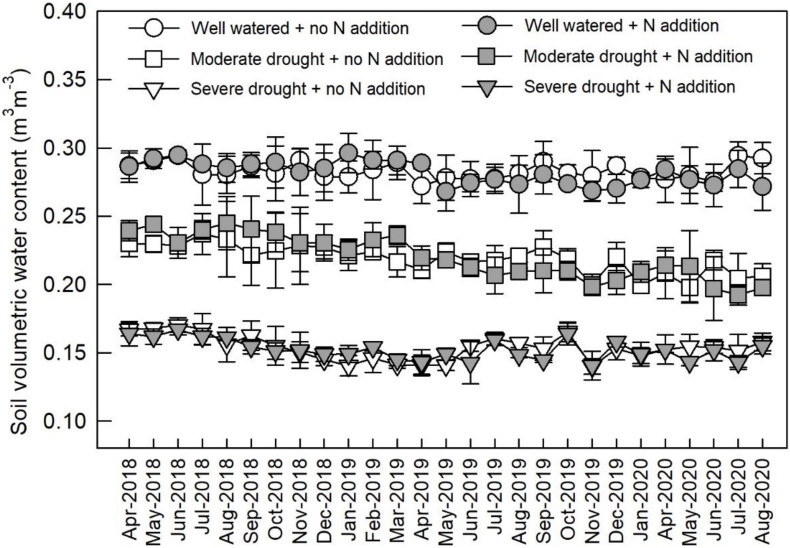
Monthly soil volumetric water content under different watering and N addition conditions. Values are means ± SE (*n* = 3).

From April 2018, the plots were divided into three watering regimes: well-watered (ca. 80% of field capacity), moderate drought (ca. 60% of field capacity), and severe drought (ca. 40% of field capacity) (Figure 1). The soil water content in each plot was measured every 3–5 days and maintained at a constant level. Within each watering regime, plots were assigned to two N addition treatments: control (0 kg N ha^–1^ year^–1^) and N addition (80 kg N ha^–1^ year^–1^). Therefore, for each species in each treatment combination of N addition and watering regime, there were three replicates. Ammonium nitrate (NH_4_NO_3_) was weighed (1.905 g) and mixed with 5 L of water, and the solution was sprayed onto the plots once every month. The control plots received an equivalent volume of deionized water.

### Growth Measurements

In August 2020, all harvested saplings were separated into leaves, stems (including branches), and roots, and then oven-dried at 70°C for 72 h to determine their biomass (g). Sapling height (cm) and basal diameter (mm) were also recorded. Sapling height was measured from the ground to the uppermost tip of the plant using a metric ruler, and basal diameter was measured near the soil surface with a digital caliper. To measure specific leaf area (*SLA*, cm^2^ g^–1^), 10 fully expanded leaves were randomly collected from each individual and measured with a leaf area meter (Licor-3100A, Li-Cor, Lincoln, NE, United States). The leaves were then oven-dried at 70°C in an oven at constant weight to determine the dry mass. *SLA* was calculated as the fresh leaf area per unit of dry mass.

### Water Relations

The predawn (Ψ_pd_, MPa) and midday (Ψ_md_, MPa) leaf water potentials were measured using a Scholander-type pressure chamber (PMS 1505D, PMS Instruments, Corvalis, Oregon, United States). The measurements were performed between 06:00 and 07:00 for Ψ_pd_ and between 12:00 and 14:00 for Ψ_md_. Three leaves from each individual sapling were sampled for the leaf water potential measurements.

### Wood Anatomy Measurements

Terminal stems were used for wood anatomical measurements. The sapwood samples (with bark and pith removed) were carefully immersed in a small graduated cylinder to obtain the fresh volume, and then oven-dried at 70°C for 72 h to determine the dry mass. Sapwood density (*WD*, g cm^–3^) was calculated as the ratio of dry mass to fresh volume.

Transverse sections of xylem tissue (∼30 μm thick) were cut using a sliding microtome (SM2010R, Leica, Nassloch, Germany). Images of the slides were photographed under a light microscope (DM2500, Leica) and analyzed using ImageJ software (National Institutes of Health, Bethesda, MD, United States). The mean hydraulically weighted vessel diameter (*D*_H_, μm) was calculated as: $D_{\text{H}}={\left[\frac{\sum D^{4}}{\text{N}}\right]}^{0.25}$ (Sterck et al., 2008), where *D* is the diameter of the vessels and N is the number of vessels in the cross-section. Vessel frequency (*VF*, average vessel numbers mm^–2^) was calculated as vessel number per relevant image area. Sapwood theoretical hydraulic conductivity (*K*_th_, kg s^–1^ m^–1^ MPa^–1^) was calculated as: $K_{\text{th}}=\frac{D_{\text{H}}^{4}\,\pi }{128\,\eta }\times V\,F\times 1,000$ (Pfautsch et al., 2016), where η represents the viscosity of the water at 20°C (1.002 × 10^–9^ MPa s).

### Leaf Anatomy Measurements

Ten fully expanded leaves from each replicate sapling were collected to determine the anatomical traits of the leaves and stomata. The leaf samples were immediately stored in a cool box with ice and transported to the lab. To measure the thicknesses of the leaf palisades (*PMT*, μm) and spongy mesophylls (*SMT*, μm), leaf transverse-sections were obtained using a rotary microtome (RM2255, Leica), and then mounted and examined at a magnification of 400 × under a light microscope (DM2500, Leica).

To determine leaf vein density (*VD*, mm mm^–2^), samples were selected from the middle of the right-hand sides of the leaves. The samples (∼1 mm^2^) were then imaged using a light microscope, and the total vein length (one to three orders) was analyzed using ImageJ. *VD* was defined as total vein length per unit leaf area.

To measure the stomata anatomy, leaf epidermises on the adaxial side were extracted centrally from the leaf midway between the midrib and the margin. Mounted sections were observed under a light microscope (DM2500, Leica) to examine stomatal density (*SD*, no mm^–2^) and length (*SL*, μm). For each leaf epidermis, 20 stomata were randomly selected to measure *SL*, and three fields (500 × 500 μm) were sampled for *SD*. The stomatal pore index (*SPI*, %) was calculated as: *SPI* = *SD* × *SL*^2^ × 10^–4^ (Sack et al., 2003).

### Non-structural Carbohydrate Assays

Oven-dried leaf and xylem samples were ground to a fine powder using a ball mill to determine the soluble sugar (ss, mg g**^–^**^1^) and starch (st, mg g**^–^**^1^) concentrations. The fine powder was weighed (20 mg per sample) and then extracted in 5 ml of 80% aqueous ethanol (v/v) in a polyethylene tube. The mixture was boiled in a water bath at 95°C for 30 min, and then centrifuged at 3,000 rpm for 5 min. The supernatant was collected and the pellet re-extracted, then boiled and centrifuged as before. The supernatants were reserved and evaporated to the last 1–3 ml in a rotational vacuum concentrator at 40°C. The ss was determined based on the supernatants colorimetrically at 620 nm using the anthrone colorimetric method (Ebell, 1969). The st was determined based on the remaining pellets after the ethanol and water were extracted and assayed enzymatically using a total starch assay kit (Megazyme International Ireland Ltd., Wicklow, Ireland).

### Statistical Analysis

Three-way ANOVAs were employed to test the primary and interactive effects of N addition, drought stress, and tree species on each of the physiological and anatomical traits (height, basal diameter, biomass, Ψ_pd_, Ψ_md_, *K*_th_, *WD*, *D*_H_, *VF*, *SMT*, *PMT*, *VD*, *SLA*, *SD*, *SL*, *SPI*, leaf ss, leaf st, xylem ss, and xylem st). Two-way ANOVAs were used to assess the effects of N addition, drought, and their interaction in each tree species. Differences between the three watering regimes for each N addition rate were evaluated using *post-hoc* with Tukey’s HSD tests. Differences between the control and N addition under each watering regime were analyzed by independent samples *t*-tests. Differences were considered to be statistically significant at *P* < 0.05. Principal component analyses (PCA) were performed using CANOCO software for Windows 4.5 (Ithaca, NY, United States). Other statistical analyses were conducted using SPSS 22.0 (SPSS Inc., Chicago, IL, United States).

## Results

### Sapling Growth

**FIGURE 2:**
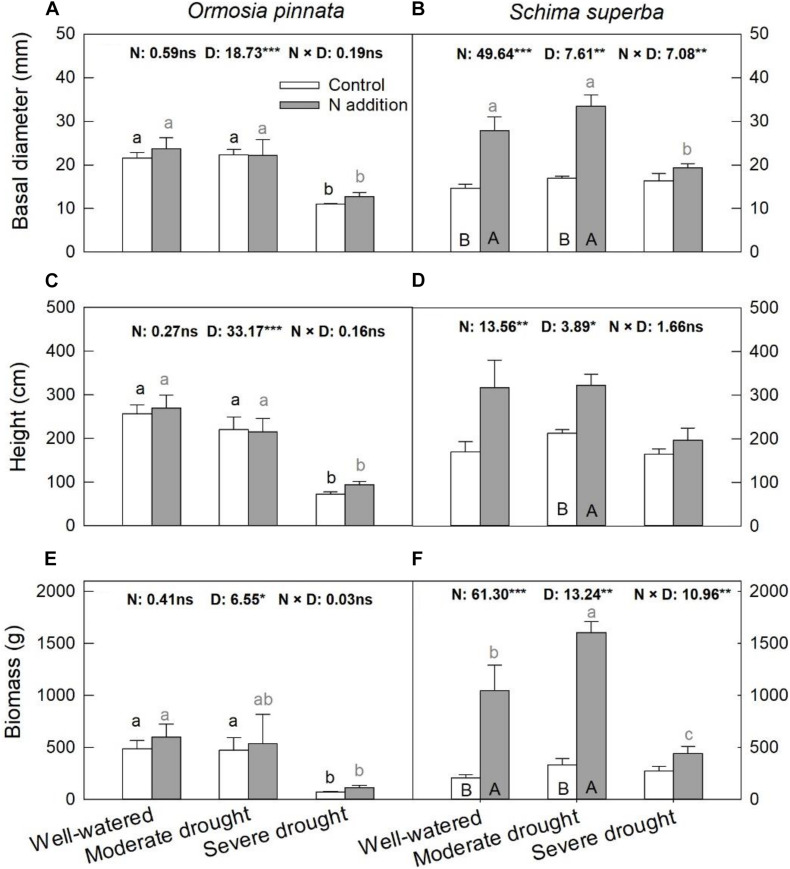
Responses of basal diameter **(A,B)**, height **(C,D)**, and biomass **(E,F)** of *O. pinnata* and *S. superba* to different watering and N addition treatments. Bars show means ± SE (*n* = 3). Different lowercase letters above bars indicate significant differences among three watering regimes within each N addition rate. Different capitalized letters below bars indicate significant differences between control and N addition within each watering regime. *F*-values of the two-way ANOVA of nitrogen (N), drought (D), and their interactions (N × D) are indicated. *P < 0.05; **P < 0.01; ***P < 0.001; ns, not significant.

Significant three-way interactive effects of drought stress, N addition, and tree species were found for basal diameter and biomass (Table 1). The two-way interactive effects between drought stress and N addition were significant for the basal diameter and biomass in *S. superba*, while this was not the case for *O. pinnata* (*P* > 0.05; Figure 2). The main effect of N addition on sapling growth was significant for *S. superba*, but not for *O. pinnata* (Figure 2 and Table 1). Specifically, N addition enhanced (*P* < 0.05) the basal diameter, height, and biomass in *S. superba* under the moderate drought condition. However, it had no effects under severe drought. The main effect of drought was significant for sapling growth in both tree species (Figure 2 and Table 1). For *O. pinnata*, severe drought significantly decreased the basal diameter, height, and biomass under both N conditions. For *S. superba*, severe drought decreased the sapling growth only under N addition. Without N addition, drought had a negligible effect on the growth of *S. superba* saplings.

**TABLE 1:** Probability values of three-way ANOVAs for the effects of N addition, drought stress, and tree species on each of the physiological and anatomical traits.

Parameters	N addition	Drought	Species	N addition × drought	N addition × species	Drought × species	N addition × drought × species
Basal diameter	**< 0.001**	**< 0.001**	**0.034**	0.080	**< 0.001**	**0.044**	**0**.**029**
Height	**0.003**	**< 0.001**	**0.014**	0.415	**0.013**	**0.023**	0.269
Biomass	**< 0.001**	**< 0.001**	**0.001**	**0.016**	**< 0.001**	0.131	**0.023**
Ψ_pd_	**< 0.001**	**< 0.001**	**< 0.001**	**< 0.001**	0.596	**< 0.001**	0.458
Ψ_md_	**< 0.001**	**< 0.001**	**0.049**	0.429	0.349	0.567	**0.002**
*WD*	0.652	**< 0.001**	**0.034**	0.683	0.297	**0.014**	0.405
*D*_H_	0.088	**< 0.001**	**< 0.001**	**< 0.001**	0.514	**< 0.001**	**0.022**
*VF*	0.542	0.180	**< 0.001**	**0.001**	0.927	0.317	**0.001**
*K*_th_	0.057	**< 0.001**	0.131	**0.012**	0.243	**0.025**	**0.049**
*SMT*	0.143	0.342	**< 0.001**	0.518	0.989	0.914	0.482
*PMT*	0.616	0.073	**< 0.001**	**0.002**	0.230	0.682	0.274
*VD*	0.236	0.100	**< 0.001**	0.365	0.068	0.569	0.733
*SLA*	0.315	**0.025**	**< 0.001**	0.771	0.477	0.226	0.630
*SD*	0.169	0.112	**< 0.001**	0.031	0.742	0.749	**0.001**
*SL*	0.151	0.195	0.605	0.290	0.379	0.446	0.718
*SPI*	**0.007**	**0.048**	**< 0.001**	0.023	0.090	0.965	**0.010**
Leaf ss	**0.037**	0.122	**0.032**	0.212	0.219	**0.026**	0.194
Leaf st	0.449	0.942	0.430	0.672	0.437	0.891	0.938
Xylem ss	0.259	**< 0.001**	**< 0.001**	**< 0.001**	0.678	**< 0.001**	**0.004**
Xylem st	0.888	**0.004**	**< 0.001**	**0.018**	0.255	**0.002**	0.066

**Significant effects are presented in bold (P < 0.05). Ψ_*pd*_, predawn leaf water potential; Ψ_*md*_, midday leaf water potential; K_*th*_, theoretical hydraulic conductivity; WD, wood density; D_*H*_, vessel diameter; VF, vessel frequency; SMT, leaf spongy mesophyll thickness; PMT, leaf palisade mesophyll thickness; VD, leaf vein density; SLA, specific leaf area; SD, stomatal density; SL, stomatal length; SPI, stomatal pore index; ss, soluble sugar; st, starch.**

### Leaf Water Potential

**FIGURE 3:**
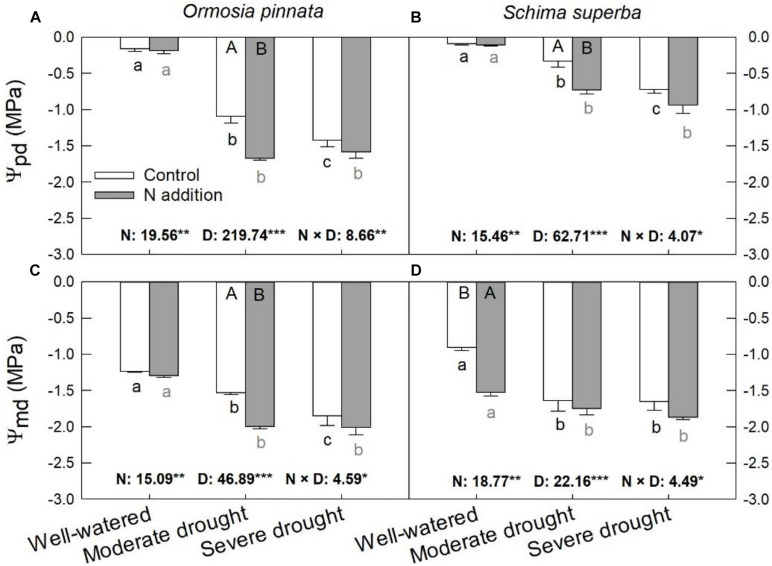
Responses of predawn (Ψ_pd_, **A,B**) and midday (Ψ_md_, **C,D**) leaf water potential of *O. pinnata* and *S. superba* to different watering and N addition treatments. Bars show means ± SE (*n* = 3). Different lowercase letters above bars indicate significant differences among three watering regimes within each N addition rate. Different capitalized letters below bars indicate significant differences between control and N addition within each watering regime. *F*-values of the two-way ANOVA of nitrogen (N), drought (D), and their interactions (N × D) are indicated. *P < 0.05; **P < 0.01; ***P < 0.001; ns, not significant.

Significant effects of N addition, drought, and their interaction on Ψ_pd_ and Ψ_md_ were observed for both tree species (Figure 3). The Ψ_pd_ and Ψ_md_ of the two species were significantly decreased (more negative) with escalating drought stress under both N conditions. Specifically, N addition decreased (*P* < 0.05) the Ψ_pd_ under moderate drought for both species, but not under the well-watered and severe drought conditions. On the other hand, N addition decreased (*P* < 0.05) Ψ_md_ under moderate drought for *O. pinnata*; the same decrease was observed under the well-watered condition for *S. superba.*

### Xylem Anatomical Traits

**FIGURE 4:**
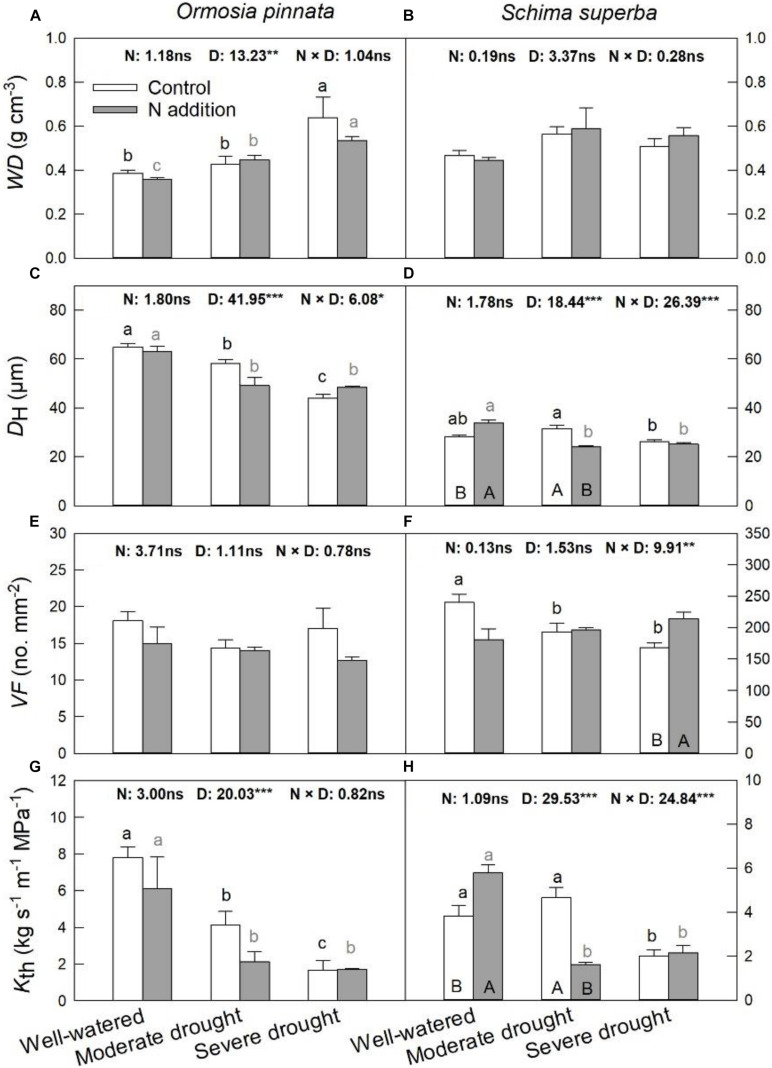
Responses of sapwood density (*WD*; **A,B**), mean hydraulically weighted vessel diameter (*D*_H_; **C,D**), vessel frequency (*VF*; **E,F**), and theoretical hydraulic conductivity (*K*_th_; **G,H**) of *O. pinnata* and *S. superba* to different watering and N addition treatments. Bars show means ± SE (*n* = 3). Different lowercase letters above bars indicate significant differences among three watering regimes within each N addition rate. Different capitalized letters below bars indicate significant differences between control and N addition within each watering regime. *F*-values of the two-way ANOVA of nitrogen (N), drought (D), and their interactions (N × D) are indicated. *P < 0.05; **P < 0.01; ***P < 0.001; ns, not significant.

*S. superba* exhibited lower (*P* < 0.05) *D*_H_ (28.31 ± 0.53 μm) and higher *VF* (240.32 ± 12.92 no. mm^–2^) than *O. pinnata* (64.82 ± 1.49 μm and 18.08 ± 1.24 no. mm^–2^ for *D*_H_ and *VF*, respectively), and had a lower *K*_th_ (3.82 ± 0.46 kg s^–1^ m^–1^ MPa^–1^) than *O. pinnata* (7.81 ± 0.57 kg s^–1^ m^–1^ MPa^–1^). Significant three-way interactive effects of drought stress, N addition, and tree species were observed for *D*_H_, *VF*, and *K*_th_ (Table 1). *WD*, *D*_H_, *VF*, and *K*_th_ of *O. pinnata* did not change with N addition under all watering regimes (Figure 4). There were significant interactive effects between N addition and drought treatments for *D*_H_, *VF*, and *K*_th_ in *S. superba*. N addition increased *D*_H_ and *K*_th_ under the well-watered condition in *S. superba*, but significantly decreased the munder moderate drought. N addition also significantly increased *VF* under the severe drought condition.

### Leaf and Stomatal Anatomical Traits

**FIGURE 5:**
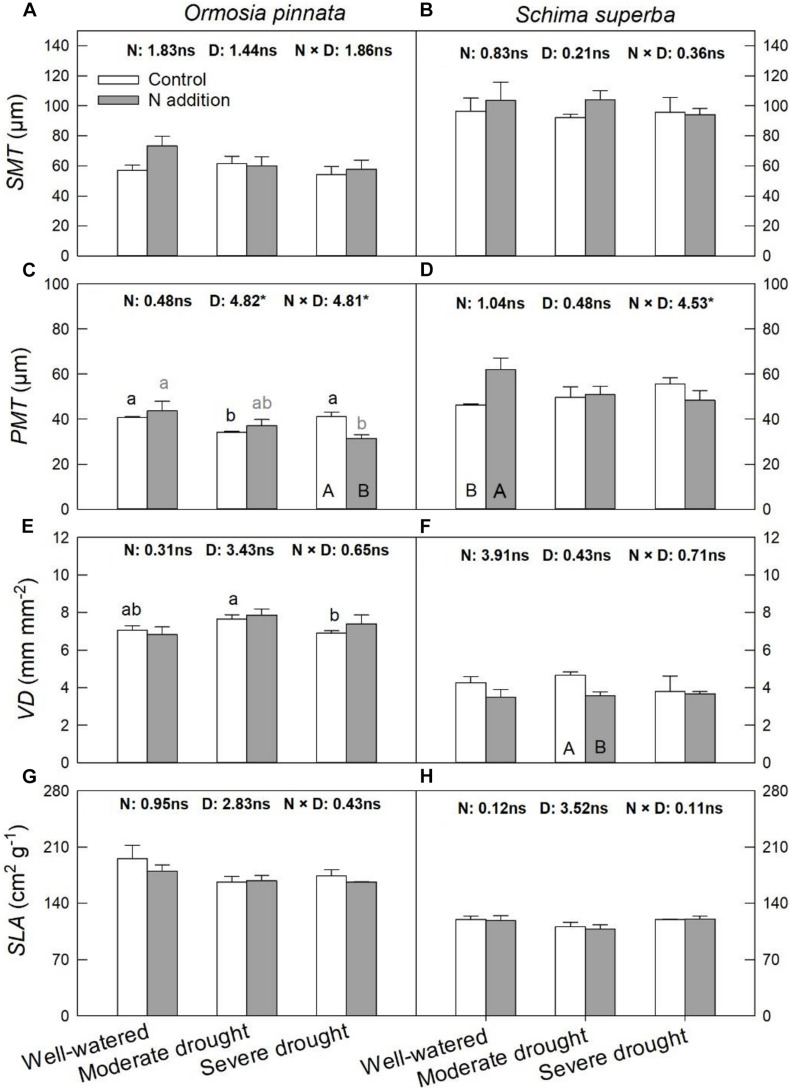
Responses of leaf palisade mesophyll thicknesses (*PMT*; **A,B**), spongy mesophyll thicknesses (*SMT*; **C,D**), leaf vein density (*VD*; **E,F**), and specific leaf area (*SLA*; **G,H**) of *O. pinnata* and *S. superba* to different watering and N addition treatments. Bars show means ± SE (*n* = 3). Different lowercase letters above bars indicate significant differences among three watering regimes within each N addition rate. Different capitalized letters below bars indicate significant differences between control and N addition within each watering regime. *F*-values of the two-way ANOVA of nitrogen (N), drought (D), and their interactions (N × D) are indicated. *P < 0.05; ns, not significant.

According to the two-way and three-way ANOVAs, N addition, drought, or their interactions with species had no significant effect on *SMT*, *VD*, and *SLA* (Figure 5 and Table 1). Significant interactive effects of N addition and drought were detected for *PMT* (Figure 5). In detail, N addition significantly decreased *PMT* under severe drought for *O. pinnata*, but increased it under the well-watered condition for *S. superba*. *SLA* significantly decreased under moderate drought in *O. pinnata*.

**FIGURE 6:**
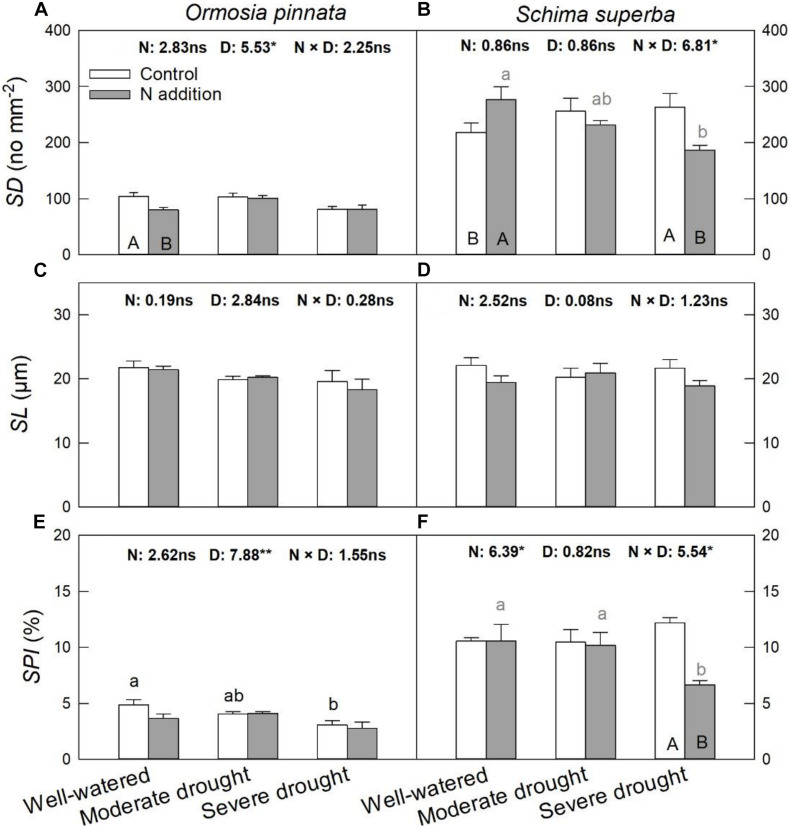
Responses of stomatal density (*SD*; **A,B**), stomatal length (*SL*; **C,D**), and stomatal pore index (*SPI*; **E,F**) of *O. pinnata* and *S. superba* to different watering and N addition treatments. Bars show means ± SE (*n* = 3). Different lowercase letters above bars indicate significant differences among three watering regimes within each N addition rate. Different capitalized letters below bars indicate significant differences between control and N addition within each watering regime. *F*-values of the two-way ANOVA of nitrogen (N), drought (D), and their interactions (N × D) are indicated. *P < 0.05; **P < 0.01; ns, not significant.

*S. superba* had higher (*P* < 0.05) *SD* (218.08 ± 17.06 no. mm^–2^) and *SPI* (10.55 ± 0.31%) than *O. pinnata* (103.78 ± 6.93 no. mm^–2^ and 4.88 ± 0.43% for *SD* and *SPI*, respectively). Under the well-watered condition, N addition decreased *SD* in *O. pinnata* but increased it in *S. superba* (Figure 6). Meanwhile, N addition decreased SD in *S. superba* under severe drought. N addition, drought, or their interaction had no significant effect on *SL* in both tree species (Figure 6 and Table 1). When devoid of N addition, drought decreased *SPI* in *O. pinnata*, but not in *S. superba*; while with N addition, *SPI* of *S. superba* decreased under severe drought.

### Non-structural Carbohydrates

**FIGURE 7:**
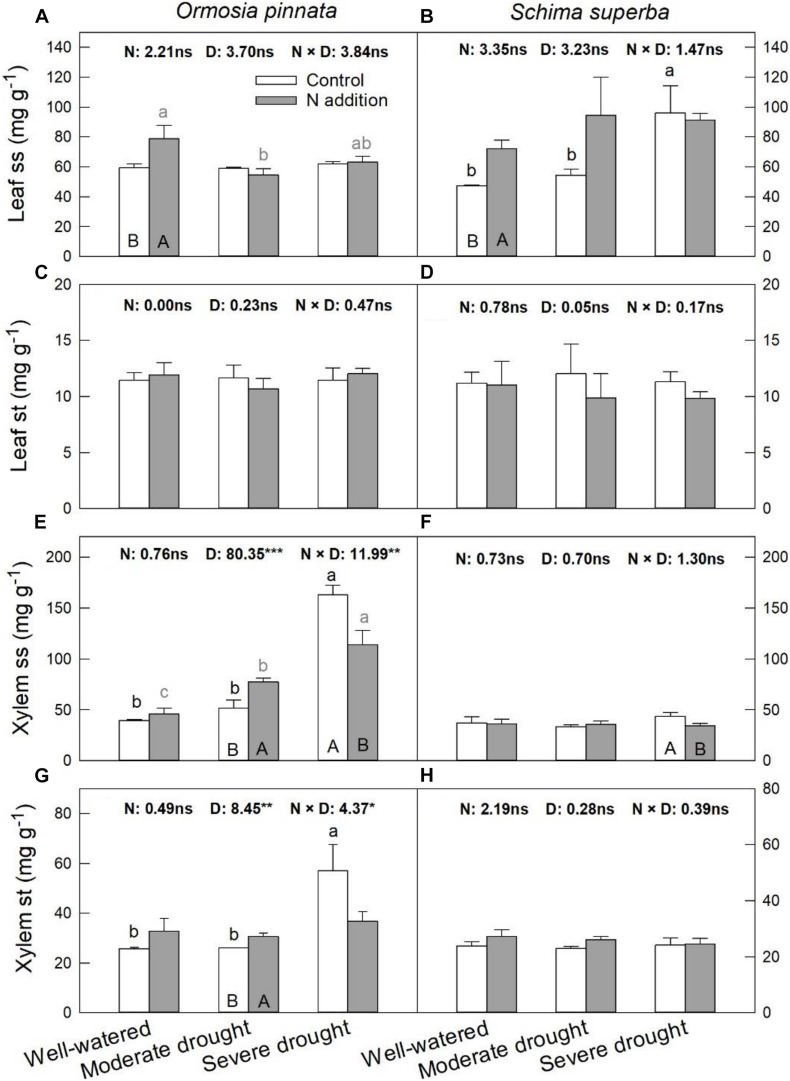
Responses of leaf soluble sugar (ss; **A,B**) and starch (st; **C,D**), xylem ss **(E,F)**, and st **(G,H)** of *O. pinnata* and *S. superba* to different watering and N addition treatments. Bars show means ± SE (*n* = 3). Different lowercase letters above bars indicate significant differences among three watering regimes within each N addition rate. Different capitalized letters below bars indicate significant differences between control and N addition within each watering regime. *F*-values of the two-way ANOVA of nitrogen (N), drought (D), and their interactions (N × D) are indicated. *P < 0.05; **P < 0.01; ***P < 0.001; ns, not significant.

Nitrogen addition increased leaf ss of both species under the well-watered condition (Figure 7). However, it had no significant effect on leaf st for both tree species under all watering regimes. Significant three-way interactive effects of drought stress, N addition, and tree species were detected for the xylem ss (Table 1). N addition significantly increased xylem ss in *O. pinnata* under moderate drought and decreased it for both tree species under severe drought. Under both N addition rates, severe drought significantly increased xylem ss in *O. pinnata*. For xylem st, N addition also increased it in *O. pinnata* under moderate drought, but had no effect on *S. superba.*

### Traits Coordination

**FIGURE 8:**
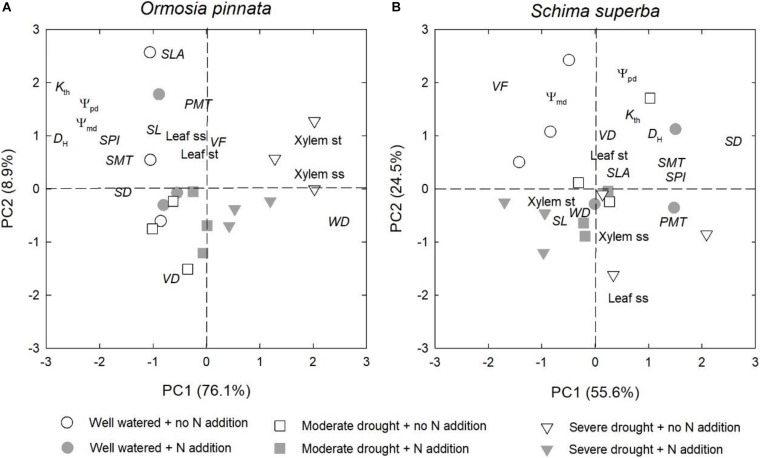
Effects of N addition and drought on the physiological and anatomical traits of *O. pinnata* **(A)** and *S. superba* **(B)** using principal component analysis (PCA). See Table 1 for trait codes.

Overall, drought significantly affected the physiological and anatomical responses for both tree species, but especially for *O. pinnata* (Figure 8 and Table 1). The physiological and anatomical traits of *O. pinnata* under the severe drought treatment were clearly distinct (*P* < 0.01) from the well-watered and moderate drought treatments on the PC1 axis. The PC1 was most strongly influenced by the xylem ss and st, *WD*, Ψ_pd_, Ψ_md_, *D*_H_, and *K*_th_. The physiological and anatomical traits of *S. superba* under the severe drought treatment were clearly distinguished (*P* < 0.05) from the well-watered treatment on the PC2 axis, and the PC2 was most strongly influenced by leaf ss, Ψ_pd_, Ψ_md_, and *K*_th_. We also found that Ψ_pd_, Ψ_md_, and *K*_th_ demonstrated a negative correlation with the xylem ss for *O. pinnata*, but showed a negative correlation with the leaf ss for *S. superba.*

However, the responses of the two species to drought were different when N addition was considered. For *O. pinnata*, drought significantly increased the PC1 scores under both N addition rates. On the contrary, for *S. superba*, drought significantly increased the PC1 scores in the control group, but decreased the PC1 scores in the N addition group. This meant that N addition altered the response trajectories of physiology and anatomy to drought. For the first axis, N addition also significantly modified the physiological and anatomical responses of the two species under the severe drought treatment, and the physiological and anatomical responses of well-watered *S. superba* (Figure 8 and Table 2).

**TABLE 2:** N addition, drought, and their interactive effects on the first and second principal component scores of the physiological and anatomical traits of two tree species.

N addition rate	Watering regime	Species			
		
		*O. pinnata*		*S. superba*	
			
		PC1	PC2	PC1	PC2
Control	Well-watered	–0.99 ± 0.12b	0.84 ± 1.61	–0.92 ± 0.47bB	1.33 ± 0.99a
	Moderate drought	–0.66 ± 0.33b	–0.83 ± 0.64	0.33 ± 0.67ab	0.53 ± 1.04ab
	Severe drought	1.78 ± 0.43aA	0.62 ± 0.64	0.86 ± 1.07aA	–0.86 ± 0.76b
N addition	Well-watered	–0.75 ± 0.18c	0.47 ± 1.14	0.99 ± 0.87aA	0.16 ± 0.83
	Moderate drought	–0.12 ± 0.18b	–0.37 ± 0.45	0.01 ± 0.33ab	–0.34 ± 0.42
	Severe drought	0.72 ± 0.42aB	–0.43 ± 0.24	–1.2 ± 0.43bB	–0.64 ± 0.50
**Analysis of variance**					
N addition		0.37	0.90	0.30	3.21
Drought		83.10***	3.46	0.32	5.35*
N addition × Drought		12.50**	0.67	12.63**	1.42

Different lowercase letters indicate significant differences among three watering regimes within each N addition rate. Different capitalized letters indicate significant differences between control and N addition within each watering regime. F-values of the two-way ANOVA of N addition, drought, and their interactions are shown. *P < 0.05; **P < 0.01; ***P < 0.001.

## Discussion

### Responses of Growth and Leaf Water Potential to Nitrogen Addition and Drought

We investigated the possibility that N addition associated with drought could differentially affect the growth and anatomical traits of the co-occurring leguminous *O. pinnata* and the non-leguminous *S. superba*. We found that patterns of growth responses to N addition and drought strongly differed between *O. pinnata* and *S. superba* rather than simply in terms of magnitude. Moreover, the effects of N addition on sapling growth were contingent upon soil water conditions. Specifically, we found that N addition translated to the enhanced growth of *S. superba* saplings under well-watered and moderate drought conditions, but failed to elicit a positive effect under severe drought. However, N addition had little effect on the growth of *O. pinnata* saplings regardless of soil water availability, which suggested that the leguminous *O. pinnata* was irresponsive to additional N inputs. Similarly, Huang et al. (2015) and Liu et al. (2017) also found a lack of growth response of *O. pinnata* to N addition (100 kg N ha^–1^ year^–1^) in Southern China. The greater responses of *S. superba* compared with *O. pinnata* to N addition in this study confirmed the hypothesis that leguminous species may lose their advantage over non-leguminous species if the availability of N increases (Xia and Wan, 2008). Without N addition, drought stress had non-significant effects on the growth of *S. superba*, but inhibited growth in *O. pinnata* under the severe drought condition. Previous studies have also reported that *S. superba* possessed high plasticity and resistance under drought conditions (Kuang et al., 2017; Duan et al., 2019). These results were consistent with the first hypothesis that the leguminous *O. pinnata* is less sensitive to N supplies, but more sensitive to drought than the non-leguminous *S. superba*.

The quantification of leaf water relations may be a useful tool for the determination of overall plant quality and stress resistance. Assuming no transpiration at predawn, Ψ_pd_ is considered the best representation of water status at the plant level (Luiz Ferraresso Conti Junior et al., 2020). In this study, the Ψ_pd_ became more negative as drought stress increased for both tree species. We also found that N addition significantly lowered leaf Ψ_pd_ and Ψ_md_ of *O. pinnata* saplings under moderate drought, and significantly decreased leaf Ψ_pd_ and Ψ_md_ of *S. superba* under different watering regimes. This partially supported the second hypothesis that excessive N deposition can aggravate the negative effects of drought on plants. A similar phenomenon has also been reported by Zhang et al. (2019) in *Quercus mongolica* seedlings that were coping with the interactions of severe drought conditions (20–30% field capacity) and N addition (23, 46, and 69 kg N ha^–1^ year^–1^).

### Responses of Xylem and Leaf Anatomical Traits to Nitrogen Addition and Drought

The two selected tree species in this study differed substantially in terms of their stem xylem anatomical traits. Compared with *O. pinnata* (*D*_H_ = 64.82 ± 1.49 μm, *VF* = 18.08 ± 1.24 no. mm^–2^), *S. superba* had narrower but more abundant stem vessels (*D*_H_ = 28.31 ± 0.53 μm, *VF* = 240.32 ± 12.92 no. mm^–2^), which suggests a relatively higher hydraulic safety and lower risk of embolism (De Micco et al., 2008). Consistent with previous studies, drought increased the *WD* and decreased the *D*_H_ and *K*_th_ of *O. pinnata* regardless of N condition, which, in turn, increased hydraulic safety and reduced the risk of embolism (Sperry et al., 2008). These adaptive changes can reduce water loss and facilitate the acclimation of plants to drought. Furthermore, it is worth noting that the effects of N addition on the xylem anatomical traits of *S. superba* were dependent on the soil water availability. Under the well-watered condition, N addition did not affect the *WD*, but induced larger *D*_H_ and lower *VF*, which facilitate greater *K*_th_ to meet higher water use and increased growth rate of N-fertilized plants. Conversely, N addition decreased the *D*_H_ and *K*_th_ under moderate drought and increased the *VF* under severe drought. These results were consistent with a meta-analysis by Borghetti et al. (2017), where they found that enhanced N availability might favor xylem structures with more vessels per surface unit rather than increased conduit size. Generally, high *VF* can be beneficial for speedy water transport and the promotion of hydraulic safety, as more vessels will remain functional at a certain rate of xylem embolism (Brodribb et al., 2012).

The hydraulic architectures of leaves are distinct from those in stems, as leaves have two major pathways for water movement, i.e., the leaf vein xylems and the bundle sheaths and mesophylls outside the xylem; with the latter pathway comprising the major constraint of water transport within leaves (Sack and Holbrook, 2006). *O. pinnata* leaves possess more compact spongy tissues and consistently higher *VD* and *SLA* compared to *S. superba* leaves. In this study, leaf anatomical traits showed less response to N addition and drought compared with stem xylem traits. For *S. superba*, N addition and drought had no significant effect on *SMT*, *VD*, and *SLA*. We found that N addition induced a higher *PMT* of *S. superba* under the well-watered condition. Thicker palisade tissues, which have been associated with greater quantities of chlorenchyma and photosynthetic capacities (Cruz et al., 2019), allowed a deeper light penetration and facilitated CO_2_ diffusion to optimize photosynthesis (Kofidis et al., 2003). N addition decreased *PMT* in *O. pinnata* under the severe drought condition, which may also relate to lower photosynthetic and growth rates in *O. pinnata* under drought conditions.

Stomatal traits are intimately correlated with gas exchange and drought resistance. Consequently, stomata emerged as an ideal model for exploring the physiological response mechanisms of drought stress in plants (Lu et al., 2020). Compared with *O. pinnata*, *S. superba* had similar size but more densely packed stomata. Previous studies reported that drought resulted in a higher stomatal density and reduced stomatal size, which together could boost the adaptation of plants to drought conditions (Xu and Zhou, 2008; Durand et al., 2020). In this study, the *SD* of *S. superba* showed a tendency to increase (but not statistically significant) with drought stress. However, *O. pinnata* had a reduced *SD* under the severe drought condition. N addition increased the *SD* of *S. superba* under the well-watered condition, but decreased the *SD* and *SPI* under severe drought. The reduced *SD* and *SPI* may lead to a decrease in the maximum stomatal conductance, which permits improved water conservation during drought (Hetherington and Woodward, 2003; Bertolino et al., 2019).

### Responses of Non-structural Carbohydrates to Nitrogen Addition and Drought

To reduce osmotic potential, plants often accumulate osmolytes such as proline, soluble sugars, and amino acids (Kuang et al., 2017; Li et al., 2020). Parallel with Ψ_pd_ and Ψ_md_, N addition and drought increased the NSC concentrations, which was evidenced by significant increases in soluble sugar and starch in the leaves or xylem for both *O. pinnata* and *S. superba*. This could also be used for the regulation of hydraulic functions under embolism risks (Zhang et al., 2021). Moreover, there were different NSC patterns in response to N addition and drought between the two selected species and organs (leaves and xylem). Firstly, soluble sugars in both the leaves and xylem of *S. superba* significantly increased under severe drought, but maintained a stable level under moderate drought. Severe drought significantly stimulated soluble sugar and starch accumulation in the xylem of *O. pinnata.* However, it had no effect on the accumulations in *O. pinnata* leaves, which indicated that the xylem has a more sensitive osmotic response to drought than leaves in *O. pinnata*. Secondly, N addition stimulated soluble sugars in the leaves and xylem of *O. pinnata* under well-watered and moderate drought conditions, respectively, but decreased xylem soluble sugar under severe drought conditions. Furthermore, we did not detect starch hydrolysis in the leaves and xylem for both species, which was evidenced by the absence of a significant decrease in starch under N addition and drought conditions.

## Conclusion

In this study, we found that the application of N had little interaction with drought in the growth of leguminous *O. pinnata* saplings. However, it led to enhanced growth in non-leguminous *S. superba* saplings under moderated drought conditions. The stimulation N addition caused in *S. superba* growth under moderate drought appeared to have a cost in terms of reduced xylem hydraulic conductance, as evidenced by a decreased vessel diameter and theoretical hydraulic conductivity. The smaller vessel diameter and higher vessel frequency of *S. superba* due to N addition under drought may have also resulted in increased resistance to drought-induced xylem embolism. N addition significantly decreased the *SD* and *SPI* of *S. superba* under severe drought, which may have led to a lower stomatal conductance; thus improving water conservation. N addition also increased xylem resident soluble sugar and starch of *O. pinnata* under moderate drought, and decreased the xylem soluble sugar under severe drought for both species. However, no effect of N addition on leaf soluble sugar or starch of drought-stressed saplings was detected, which indicated variable strategies at the organ level for coping with the combined effects of N addition and drought. The results suggest that the effects of N addition on tree growth and anatomical traits depended on water conditions. Furthermore, leguminous *O. pinnata* may be less sensitive to N supplies but more sensitive to drought than non-leguminous *S. superba*.

## Data Availability Statement

The raw data supporting the conclusions of this article will be made available by the authors, without undue reservation.

## Author Contributions

YL and XF conceived this study. ZW, HL, and CZ conducted the experiment. YL wrote the main manuscript. All authors contributed to writing and editing the manuscript.

## Conflict of Interest

The authors declare that the research was conducted in the absence of any commercial or financial relationships that could be construed as a potential conflict of interest.

## Publisher’s Note

All claims expressed in this article are solely those of the authors and do not necessarily represent those of their affiliated organizations, or those of the publisher, the editors and the reviewers. Any product that may be evaluated in this article, or claim that may be made by its manufacturer, is not guaranteed or endorsed by the publisher.
